# The influence of atrial fibrillation on the mortality of incident ESRD patients undergoing maintenance hemodialysis

**DOI:** 10.1371/journal.pone.0228405

**Published:** 2020-01-30

**Authors:** Hui-ling Hsieh, Shih-chang Hsu, Ho-shun Cheng, Chun-you Chen, Wen-cheng Huang, Yuh-mou Sue, Feng-yen Lin, Chun-ming Shih, Jaw-wen Chen, Shing-jong Lin, Po-hsun Huang, Chung-te Liu

**Affiliations:** 1 Division of Nephrology, Department of Internal Medicine, Wan Fang Hospital, Taipei Medical University, Taipei, Taiwan; 2 Graduate Institute of Medical Science, National Defense Medical Center, Taipei, Taiwan; 3 Emergency Department, Department of Emergency and Critical Medicine, Wan Fang Hospital, Taipei Medical University, Taipei, Taiwan; 4 Department of Emergency Medicine, School of Medicine, College of Medicine, Taipei Medical University, Taipei, Taiwan; 5 Division of Cardiology, Department of Internal Medicine, Wan Fang Hospital, Taipei Medical University, Taipei, Taiwan; 6 Department of Radiation Oncology, Wan Fang Hospital, Taipei Medical University, Taipei, Taiwan; 7 Graduate Institute of Clinical Medicine, College of Medicine, Taipei Medical University, Taipei, Taiwan; 8 Department of Internal Medicine, School of Medicine, College of Medicine, Taipei Medical University, Taipei, Taiwan; 9 Division of Cardiology and Cardiovascular Research Center, Department of Internal Medicine, Taipei Medical University Hospital, Taipei, Taiwan; 10 Division of Cardiology, Department of Medicine, Taipei Veterans General Hospital, Taipei, Taiwan; 11 Cardiovascular Research Center, National Yang-Ming University, Taipei, Taiwan; 12 Department of Medical Research, Taipei Veterans General Hospital, Taipei, Taiwan; 13 Institute of Pharmacology, National Yang-Ming University, Taipei, Taiwan; 14 Institute of Clinical Medicine, National Yang-Ming University, Taipei, Taiwan; 15 Board of Directors, Taipei Medical University, Taipei, Taiwan; Kaohsiung Medical University Hospital, TAIWAN

## Abstract

**Background:**

Atrial fibrillation (AF) is highly prevalent, occurring in 1%–2% of the adult population, increasing the risk of stroke, and resulting in considerable healthcare costs. While stroke is a major complication of AF, end-stage renal disease (ESRD) patients also have a high risk of stroke, suggesting that AF is a possible risk factor for mortality of ESRD patients. However, whether the existence of AF at the initiation of hemodialysis predicts higher mortality risk of incident ESRD patients remains to be defined.

**Methods:**

This retrospective cohort study was performed at Wanfang Hospital from January 2004 to May 2018. The end points were mortality of patients or the end of the study. Incident ESRD patients who were on maintenance hemodialysis for more than 3 months were eligible for inclusion. Cox proportional regression and Kaplan–Meier survival curves were used to determine the association between predictors and mortality. The association between AF and echocardiographic parameters, causes of death were also investigated.

**Results:**

Of the 393 incident ESRD patients at initiation of hemodialysis, 57 (14.5%) had AF and the median age was 71 years. Patients with AF were significantly older; showed significantly higher C-reactive protein levels, more heart failure, chronic obstructive pulmonary disease and mortality. Multivariate Cox regression showed that AF had a hazard ratio of 4.1 (95% confidence interval: 2.4–7.0) for mortality. Age-specific analysis showed that AF was significantly associated with mortality in all age groups. Echocardiography measurements including ejection fraction and left ventricular hypertrophy (LVH) were similar in AF and non-AF patients. Cause-specific analysis showed that AF significantly associated with overall cardiovascular death and death due to acute myocardial infarction/coronary artery disease and sepsis.

**Conclusions:**

AF at the initiation of hemodialysis predicts higher mortality risk of incident ESRD patients regardless of age. The systolic function and degree of LVH were similar in AF and non-AF patients. The association between AF and sepsis-related death suggested the role of systemic inflammation on the pathogenesis of AF.

## Introduction

Atrial fibrillation (AF) is a common and complicated cardiac arrhythmia characterized by rapid and irregular atrial activation [1–3], which has a prevalence of around 1%–2% in the general population [4–6]. For the most part, AF remains silent and asymptomatic [7, 8]. Occasionally, the ventricular response becomes rapid and causes decompensated heart failure [9]. Regardless of the ventricular rate or the symptoms caused by AF, its disorganized atrial activation results in atrial thrombosis and increased risk of stroke [7–9]. In 2010, the number of annual incident cases was estimated at nearly 5 million [10]. With this high incidence, AF-related burden increased by 18.8% in men and 18.9% in women from 1990 to 2010 [10], leading to significantly increased cost of health care [9, 11–13]. Therefore, the management of AF has been an important issue.

In patients with end-stage renal disease (ESRD), the prevalence of AF is substantially higher than that of the general population, reaching as high as 3%–26.5% [14]. Furthermore, patients on hemodialysis have a higher incidence of AF, suggesting that uremia is associated with the pathogenesis of AF [15–16]. The annual report by the US Renal Data System stated that, in patients with CKD, the overall prevalence of cerebrovascular accident (CVA) was 16.1% and the 2-year survival of stage 4–5 CKD patients with CVA was 64.1%, suggesting a significant role of CVA in the outcome in this population [17]. Moreover, cardiovascular death is one of the leading causes of mortality among patients with ESRD [18–19]. These findings suggest that AF may have a causative role in the mortality of patients with incident ESRD, which remains to be defined to date.

As such, AF is a potential risk factor of mortality in patients with incident ESRD [20–22]. Nevertheless, whether AF predicts the mortality of incident ESRD patients remains to be clarified. To that end, we conducted a retrospective, longitudinal cohort study to investigate the predictive role of AF on the mortality of incident ESRD patients at the initiation of maintenance hemodialysis.

## Materials and methods

### Study design and subjects

The present study aimed to include incident ESRD patients on maintenance hemodialysis. Incident hemodialysis patients at Wan Fang Hospital, Taipei Medical University between January 2004 and May, 2018 were assessed for enrollment. Patients receiving maintenance hemodialysis, which was defined as those received maintenance hemodialysis for more than 3 months, were eligible for inclusion. Patients less than 20 years of age were excluded from analysis. This study was approved by the ethics committee and Institutional Review Board of Taipei Medical University (N201902034). Informed consent was waived by the ethics committee. The entire study was performed in accordance with the principles of the Declaration of Helsinki, as revised in 2000.

### Measurement of covariates and outcomes

Baseline laboratory data and demographic profiles obtained at the initiation of maintenance hemodialysis were used for analysis. The diagnosis and the indication of hemodialysis was according to the discretion of the attending nephrologist. Other comorbidities were defined according to the International Classification of Disease, 10^th^ Revision, clinical modification codes in the discharge diagnosis of the indexed hospitalization or clinic visiting. AF and HF were defined by the diagnosis on medical records made by cardiologists. Notably, patients with both paroxysmal and persistent AF were considered as AF patients in the present study.

The outcome was all-cause mortality. The period of mortality was defined by the time from hemodialysis initiation to mortality. The cause of death was based on the diagnosis of the indexed discharge summary made by the attending physician. Echocardiography was performed at baseline to evaluate ejection fraction (EF) and left ventricular hypertrophy (LVH). Echocardiographic diagnostic criteria of LVH were defined by the following guidelines: (1) normal heart size was defined as left ventricular mass (LVM) ≤ 115 g/m^2^ in men and ≤ 95 g/m^2^ in women and relative wall thickness (RWT) < 0.42; (2) concentric hypertrophy was defined as a LVM > 115 g/m^2^ in men and > 95 g/m^2^ in women and RWT > 0.42; (3) eccentric hypertrophy was defined as LVM > 115 g/m^2^ in men and > 95 g/m^2^ in women and RWT < 0.42; and (4) concentric remodeling was defined as LVM ≤ 115 g/m^2^ in men and ≤ 95 g/m^2^ in women and RWT > 0.42.^20^

### Statistical analysis

Continuous variables with normal distribution were reported as mean ± standard deviation, while continuous variables deviated from normal distribution were expressed as medians (25^th^ and 75^th^ percentiles). Categorical variables were reported as frequency and percentage. Comparisons of continuous variables were made by using a two-tailed t-test for unpaired samples or non-parametric methods, as appropriate. Comparisons of categorical variables were made using chi-squared test. The risk factors for mortality were analyzed using Cox proportional regressions and Kaplan-Meier (K-M) survival curve analysis. For multivariate Cox proportional regression, the potential risk factors of mortality were analyzed using univariate Cox proportional regression. Those with p values ≤ 0.2 in the univariate Cox proportional regression were included into the multivariable model. The association between predictors and outcomes in the Cox proportional regression model was expressed as hazard ratio (HR) and 95% confidence interval (CI). Statistical analysis was performed using SAS 9.4 (SAS Institute Inc., Cary, NC, USA).

## Results

### Demographic and laboratory characteristics

Among the 393 incident ESRD patients, the median follow-up period was 46.6 months and the median age of the study subjects was 71 years. Overall, 200 (50.9%) patients were male and 57 (14.5%) patients had AF. By the end of the study, 138 (35.1%) patients had died. Patients with AF were older, of more male gender and more likely to have heart failure (HF), valvular heart disease (VHD), chronic obstructive pulmonary disease (COPD) and thyroid disease. More AF patients were using aspirin, while the use of clopidogrel and warfarin were not significantly different between AF and non-AF patients. Furthermore, patients with AF presented with significantly lower creatinine, higher Na, higher aspartate aminotransferase (AST), higher C-reactive protein (CRP) levels and higher circulating white blood cell (WBC) counts. The AF patients had insignificantly lower serum phosphorus levels and higher alanine aminotransferase (ALT) levels. Numbers of patients with chronic liver disease, CVA, diabetes mellitus, as well as serum levels of albumin, K, Ca, and parathyroid hormone, transferrin saturation, hemoglobin and platelets were similar between the groups. Notably, AF patients had significantly more mortality at the end of study, suggesting that AF is a potential risk factor for mortality of incident ESRD patients (Table 1).

**Table 1:** Baseline demographic and laboratory characteristics of the study population.

Character	Total (n = 393)	Non-AF (n = 336)	AF (n = 57)	*P-*value
**Male (%)**	200 (50.9%)	169 (50.3%)	31 (54.4%)	0.55
**Age (years)**	71(57, 79)	69 (57, 78)	77 (71, 84)	<0.01^a^
**HF (%)**	216 (55%)	175 (52.1%)	41(71.9%)	<0.01
**VHD (%)**	29(7.38%)	18(5.36%)	11(19.30%)	<0.01
**CLD (%)**	73 (18.6%)	63 (18.8%)	10 (17.5%)	0.83
**COPD (%)**	26 (6.6%)	15 (4.5%)	11 (19.3%)	<0.01
**CVA (%)**	91 (23.2%)	79 (23.5%)	12 (21.1%)	0.68
**DM (%)**	259 (66%)	217 (64.6%)	42 (73.7%)	0.18
**Thyroid disease (%)**	14 (3.56%)	9(2.68%)	5(8.77%)	0.04
**Survival duration (month)**	46.6(25.1, 74.4)	52.2(28.7, 80.2)	18.7(7.5, 35.3)	< .001
**Mortality (%)**	138 (35.1%)	110 (32.7%)	28 (49.1%)	0.02
**Aspirin (%)**	54 (13.74%)	39(11.61%)	15(26.32%)	<0.01
**Clopidogrel (%)**	20 (5.09%)	16(4.76%)	4(7.02%)	0.51
**Warfarin (%)**	2 (0.51%)	2(0.6%)	0	0.56
**BUN (mg/dL)**	110.6±50.2	111.7±49.7	104.3±53.2	0.31
**Cr (mg/dL)**	9.1 (6.7, 11.7)	9.5 (7.0, 12.4)	7.3 (4.5, 8.7)	<0.01^a^
**eGFR (mL/min/1.73 m**^**2**^**)**	4.6 (3.5, 6.8)	4.4 (3.4, 6)	6.5 (4.8, 8.9)	<0.01^a^
**Albumin (g/dL)**	3.3±0.6	3.3±0.6	3.2±0.6	0.81
**Na (mmol/L)**	135.1±6.3	134.7±5.8	137±8.0	0.04
**K (mmol/L)**	4.4 (3.8, 5.0)	4.4(3.8, 4.9)	4.5 (4.1, 5.1)	0.25^a^
**Ca (mg/dL)**	7.9±1.6	7.9±1.2	7.9±1.0	0.92
**P (mg/dL)**	5.9 (4.6, 7.6)	6(4.6, 7.8)	5.4 (4.2, 6.5)	0.08^a^
**PTH (pg/mL)**	219 (94.5, 365)	219 (94.2, 362)	207 (94.5, 428)	0.83^a^
**AST (U/L)**	23 (17, 34)	22 (17, 32)	26 (20, 44)	<0.01^a^
**ALT (U/L)**	16 (12, 23)	16 (11,23)	19 (13, 29)	0.09^a^
**Ferritin (ng/mL)**	296.6(156.3, 551.9)	286.8 (144.5, 547.3)	375 (174.2, 635.7)	0.34^a^
**TSAT (%)**	0.20 (0.15, 0.28)	0.20 (0.15, 0.28)	0.20 (0.15, 0.26)	0.70^a^
**CRP (mg/dL)**	2.2 (0.5, 8.0)	1.7 (0.5,6.5)	6.1 (2.6,13.2)	<0.01^a^
**Hemoglobin (g/dL)**	8.5±1.7	8.5±1.7	8.8±1.8	0.15
**MCV (μm**^**3**^**)**	90.5 (86.8, 93.9)	90.5 (86.8, 93.8)	90.3 (86.5, 95.5)	0.92^a^
**WBC (10**^**3**^**/μL)**	8.2 (6.1, 10.5)	8.0 (5.9, 10.4)	9.0 (7.4, 11.0)	0.02^a^
**Neutrophil (%)**	77±12.2	77.0±12	76.6±13.5	0.78
**Platelet (10**^**3**^**/μL)**	172 (126, 231)	173 (128.5, 233)	163 (115, 223)	0.56^a^

AF, atrial fibrillation; HF, heart failure; VHD, Valvular heart disease; CLD, Chronic Liver Disease; COPD, chronic obstructive pulmonary disease; CVA, cerebrovascular accident; DM, diabetes mellitus; BUN, blood urea nitrogen; Cr, creatinine; eGFR, estimated glomerular filtration rate; PTH, Parathyroid hormone; AST, aspartate transaminase; ALT, alanine transaminase; TSAT, transferrin saturation; CRP, C-reactive protein; MCV, mean corpuscular volume; WBC, white blood cell. ^a^Compared using the Exact Wilcoxon two-sample test

### AF and risk of mortality in incident ESRD patients

**Fig 1:**
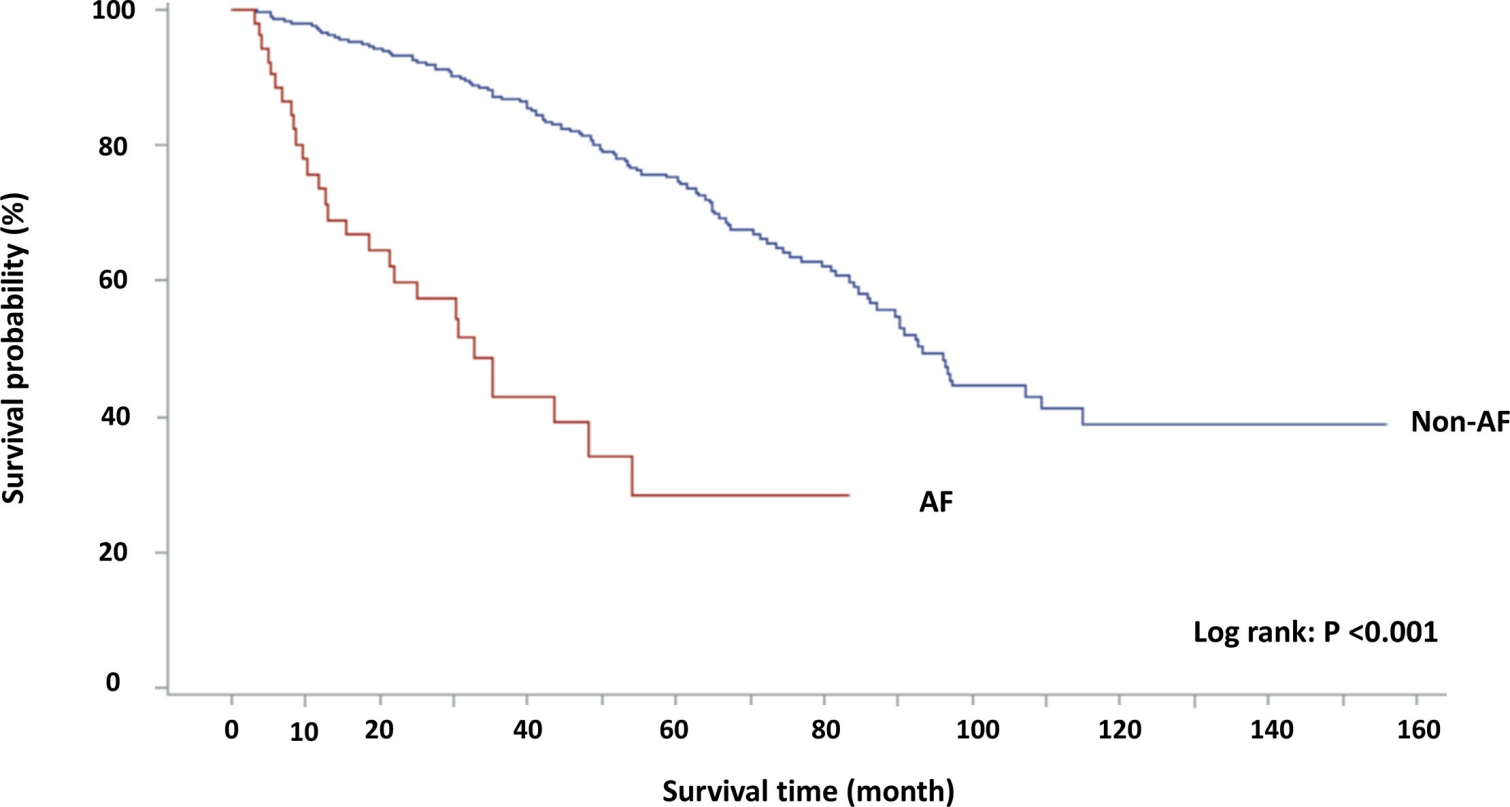
Distribution of AF patients stratified by age tertiles. Statistics was performed using the Cochrane-Armitage test. The number of AF patients were 9 (6.82%), 20 (14.18%), and 28 (23.33%) in the age tertiles of age ≤ 61, 61 < age ≤ 77, and 77 < age, respectively. AF, atrial fibrillation.

To investigate the association between AF and mortality in incident ESRD patients, AF and other potential risk factors were assessed by univariate Cox proportional regression and the predictors showing P values < 0.2 were enrolled into the multivariate analysis. Notably, serum creatinine level did not strongly correlate with residual renal function and was not included in the survival analysis. Higher WBC count signifies inflammation resembling CRP levels and was not included to avoid co-linearity of the regression model. Multivariate Cox proportional regression showed that age, CRP, presence of HF, COPD, and AF were significantly associated with mortality (Table 2). To confirm the influence of AF on mortality in incident ESRD patients, Kaplan-Meier (K-M) survival curve was used. This showed that AF patients had significantly higher mortality compared with non-AF patients (Fig 1). These findings showed that AF independently predicted mortality in incident ESRD patients.

**Table 2:** Risk factors for mortality in incident hemodialysis patients^a^.

Variable	Univariate			Multivariate		
	HR	95% CI	P value	HR	95% CI	*P*-value
**Age (per 10-year increment)**	1.8	1.6–2.2	<0.01	1.6	1.4–1.9	<0.01
**Na (per 10 mmol/L increment)**	1.1	0.8–1.5	0.49			
**P (per 5 mg/dL increment)**	0.7	0.5–1.1	0.14	1.2	0.7–2.0	0.47
**AST (per 10 U/L increment)**	1.0	0.9–1.0	0.56			
**Hemoglobin**	1.0	0.9–1.2	0.38			
**CRP (per 10 mg/dL increment)**	1.3	1.1–1.7	0.03	1.4	1.1–1.9	0.01
**Aspirin**	1.2	0.9–2.1	0.52			
**HF**	1.8	1.3–2.7	<0.01	1.7	1.1–2.7	0.01
**COPD**	3.7	2.2–6.5	<0.01	2.3	1.2–4.5	<0.01
**DM**	1.1	0.8–1.6	0.64			
**VHD**	1.5	0.7–3.0	0.22			
**Thyroid disease**	3.5	1.6–7.4	<0.01	2.2	0.8–5.7	0.11
**AF at baseline**	4.6	2.8–7.1	<0.01	4.1	2.4–7.0	<0.01

HR, hazard ratio; P, phosphorus; PTH, Parathyroid hormone; AST, aspartate transaminase; ALT, alanine transaminase; HF, heart failure; COPD, chronic obstructive pulmonary disease; DM, diabetes mellitus; VHD, Valvular heart disease AF, atrial fibrillation.

^a^by multivariate Cox proportional regression

### Age and the impact of AF on the risk of mortality

**Fig 2:**
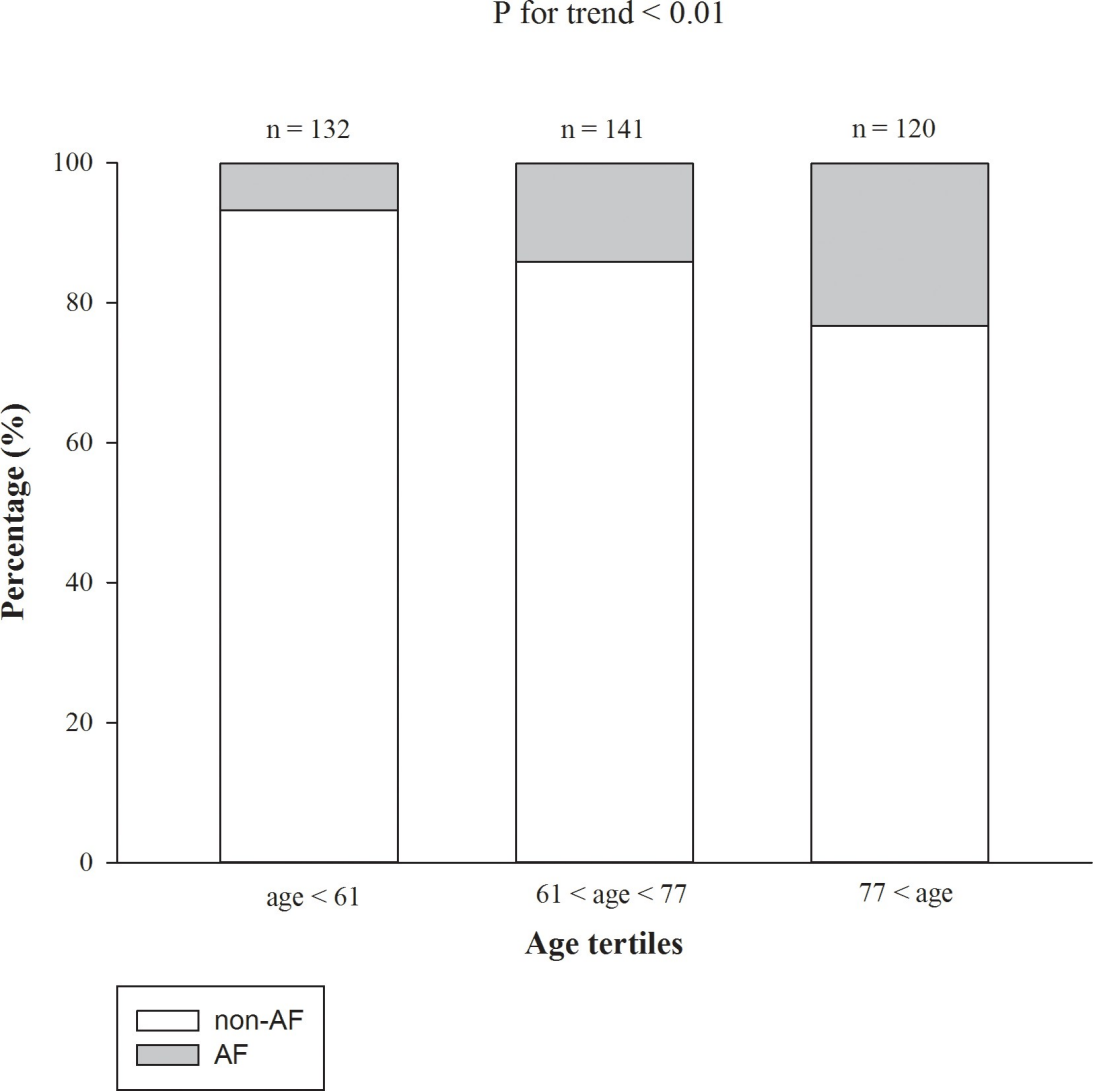
HR of AF for mortality of incident hemodialysis patients stratified by age. Multivariate model was adjusted for presence of heart failure, chronic obstructive pulmonary disease, serum levels of albumin and phosphorus. HR, hazard ratio; CI, confidence interval; AF, atrial fibrillation.

**Fig 3:**
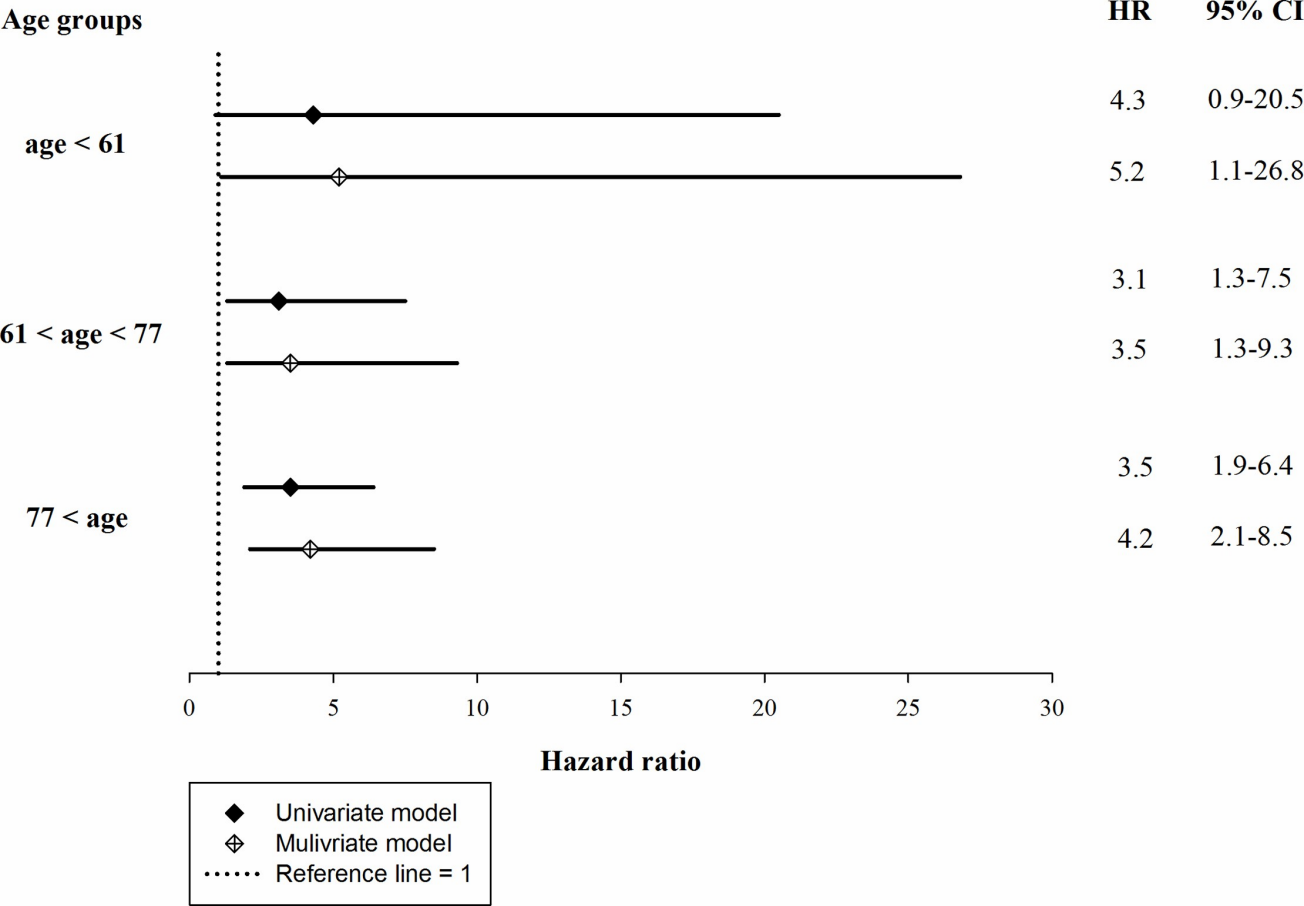
Survival probability of incident ESRD patients stratified by the presence of AF. By Kaplan-Meier method. ESRD, end-stage renal disease; AF, atrial fibrillation.

To evaluate possible confounding effects of age on the association between AF and mortality, the patients were stratified by age into tertiles for analysis. The tertiles were ≤ 61 years, > 61 to ≤ 77 years, and > 77 years. The Cochrane-Armitage test showed that the oldest tertile had significantly more AF patients (Fig 2). However, Cox proportional regression analysis showed that AF was significantly associated with mortality in all age tertiles. This finding indicated that AF predicted the mortality in incident ESRD patients regardless of age (Fig 3).

### Echocardiographic measurements in AF and non-AF incident ESRD patients

To investigate the underlying cause of the association between AF and mortality, pre-dialytic echocardiographic measurements were compared between AF and non-AF patients. Notably, in this part of analysis, only 205 patients with available echocardiographic reports were included. In the AF patients, 26 (53.06%) patients presented atrial fibrillation rhythm at the time of echocardiography examination. Overall, the echocardiography showed increased LVM and RWT, preserved EF, normal systolic and diastolic left ventricular diameter. The echocardiographic measurements were not significantly different between AF and non-AF patients. These findings suggested that both groups exhibited LVH with preserved systolic function. Nonetheless, since atrial emptying/contraction ratio was not able be measured in AF patient, diastolic dysfunction was not able to be evaluated in this group of patients. Regarding the type of LVH, AF patients showed more concentric LVH and less eccentric LVH compared to non-AF patients. Nonetheless, the distribution of the type of LVH were not significantly different between the two groups (Table 3).

**Table 3:** Pre-dialytic echocardiographic profiles of incident hemodialysis patients.

Measurement (ref.)	Total (n = 205)	Non-AF (n = 156)	AF (n = 49)	*P*-value
**Rhythm**				
Sinus rhythm (%)	179(87.32%)	156(100%)	23(46.94%)	< .001
AF rhythm (%)	26 (12.68%)	0	26(53.06%)	< .001
**IVS (6–12 mm)**	13.1±2.3	13±2.3	13.3±2.1	0.43
**LVEDD (36–52 mm)**	48.8±7.9	48.9±8.0	48.5±7.9	0.72
**LVPW (6–12 mm)**	12.9±2.3	12.8±2.3	13.2±2.2	0.25
**LV mass (67–162 g)**	251.9 (195.3, 317)	252.7 (188.7, 313.5)	246.1 (209.3, 326)	0.7^a^
**EF (≥55%)**	67 (58, 73)	67 (58, 73)	66 (56, 71)	0.59^a^
**LVESD (20–36 mm)**	31.2±8.6	31.2±8.7	31.4±8.2	0.84
**RWT (0.22–0.42 cm)**	0.52 (0.46, 0.61)	0.53 (0.46, 0.61)	0.52 (0.47, 0.6)	0.59^a^
**LVMI (43–95 g/m**^**2**^**)**	169.7±57	169.1±58.2	171.5±53.6	0.80
**BSA (n/a m**^**2**^**)**	1.5±0.2	1.5±0.2	1.5±0.2	0.76
**LVH by echocardiography**				
**Normal**	5 (2.4%)	4 (2.6%)	1 (2%)	1.00
**Concentric hypertrophy**	160 (77.7%)	120 (76.4%)	40 (81.6%)	0.43
**Eccentric hypertrophy**	26 (12.6%)	22 (14%)	4 (8.2%)	0.33
**Concentric remodeling**	15 (7.3%)	11 (7%)	4 (8.2%)	0.75

Ref, reference range; AF, atrial fibrillation; IVS, inter-ventricular septum; LVEDD, left ventricular end diastolic diameter; LVPW, left ventricular Posterior wall; LV mass, left ventricular mass; EF, ejection fraction; LVESD, left ventricular end systolic diameter; RWT, relative wall thickness; LVMI, left ventricular mass index; BSA, body surface area; LVH, left ventricular hypertrophy.

^a^Compared using the Exact Wilcoxon two-sample test.

### AF and cause of death in patients on maintenance hemodialysis

**Fig 4:**
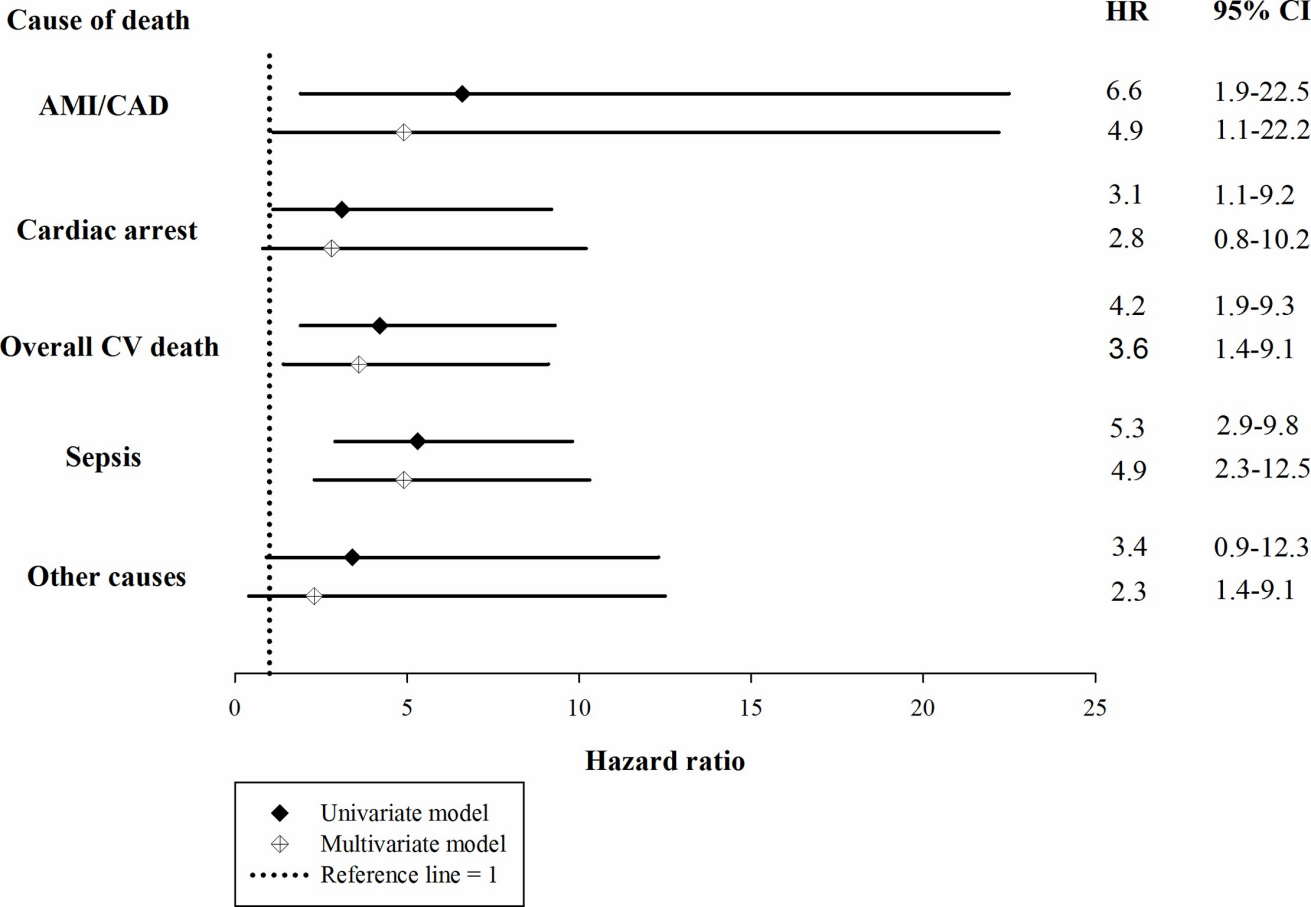
HR of AF for cause-specific mortality of incident ESRD patients on maintenance hemodialysis. Multivariate model was adjusted for age, presence of heart failure, chronic obstructive pulmonary disease, serum levels of C-reactive protein, albumin and phosphorus. HR, hazard ratio; CI, confidence interval; AF, atrial fibrillation; AMI, acute myocardial infarction; CAD, coronary artery disease; CV, cardiovascular.

Of the 138 patients who had died by the end of study, the leading cause of death was sepsis (47.8%), followed by sudden cardiac arrest (28.2%) and acute myocardial infarction (AMI)/coronary artery disease (CAD, 9.4%). Among the causes of sepsis-related death, pneumonia was the most frequent type of infection, which was followed by soft-tissue infection and catheter infection. Notably, the number of death events due to CVA were similar in both group of patients (Table 4). The association between AF and mortality due to specific causes were examined using Cox proportional regression. In the multivariate Cox proportional regression (adjusted for age, presence of HF and COPD, serum albumin, Na and phosphorus), AF was significantly associated with overall cardiovascular death, death due to AMI/ CAD and sepsis (Fig 4).

**Table 4:** Causes of death in patients on maintenance hemodialysis.

Cause of death	Total (n = 138)	Non-AF (n = 110)	AF (n = 28)
**AMI/CAD**	13 (9.4%)	9 (8.2%)	4 (14.3%)
**Sudden cardiac arrest**	39 (28.2%)	33 (30%)	6 (21.4%)
**Sepsis**	66 (47.8%)	51 (46.4%)	15 (53.6%)
**Pneumonia**	41 (29.7%)	30 (27.3%)	11 (39.3%)
**Catheter infection**	6 (4.3%)	5 (4.5%)	1 (3.6%)
**Soft-tissue infection**	9 (6.5%)	7 (6.4%)	2 (7.1%)
**Intra-abdominal infection**	4 (2.9%)	4 (3.6%)	0
**Renal necrosis**	1 (0.7%)	1 (0.9%)	0
**Urinary tract infection**	2 (1.4%)	2 (1.8%)	0
**Infective endocarditis**	2 (1.4%0	2 (1.8%)	0
**Other causes**^**a**^	20 (14.4%)	17 (15.5%)	3 (10.7%)
**Trauma**	1 (0.7%)	1 (0.9%)	0
**Hypovolemic shock**	4 (2.9%)	3 (2.7%)	1 (3.6%)
**Heart failure**	2 (1.4%)	2 (1.8%)	0
**Cerebrovascular events**	5 (3.6%)	4 (3.6%)	1 (3.6%)
**Malignancy**	3 (2.2%)	3 (2.7%)	0
**Hyperkalemia**	5 (3.6%)	3 (2.7%)	2 (7.1%)

AF, atrial fibrillation; AMI, acute myocardial infarction; CAD, coronary artery disease.

^a^trauma, hypovolemic shock, congestive heart failure, cerebrovascular events, malignancy and hyperkalemia.

## Discussion

The main finding of the present study is that AF predicts the mortality in incident ESRD patients on maintenance hemodialysis. While AF may deteriorate heart failure, echocardiographic measurements, including LVM, RWT, and EF were similar between AF and non-AF patients in our study. Finally, in addition to increased risk cardiovascular death, AF was also associated with increased risk of sepsis-related death, suggesting the role of inflammation on the pathogenesis of AF.

One study based on US Renal Data System from 1992 to 2006 had demonstrated that the prevalence of AF among hemodialysis patients increased from 3.5% to 10.7% [23]. Another Taiwanese retrospective cohort study of 15,947 patients in 2016 showed that the incidence of AF was higher among hemodialysis patients [24]. In another study of 1,130 ESRD patients conducted in 2017, 26.9% of the cohort were noted to have AF during the study period [25]. In the present cohort, 14.5% of the incident ESRD patients had AF at the initiation of hemodialysis, confirming the high prevalence and incidence of AF in dialysis population. These findings suggest that uremia may have a role in the pathogenesis AF, but the mechanism remains to be investigated.

While a previous 9-year cohort study in Japan showed that DM was highly associated with all-cause mortality in patients undergoing hemodialysis (adjusted HR: 2.39; p < 0.001) [26], the present study showed that DM was not significantly associated with the risk of mortality. This discrepancy may result from the difference in race, glucose control status or relative short study period of the present study.

In the non-dialysis population, the incidence of stroke in AF patients ranges 5.9–9.0 per 100 patient-years [27]. In contrast, a large cohort study based on data from the international Dialysis Outcomes and Practice Patterns Study (DOPPS) showed that in hemodialysis patients with AF, the incidence of stroke was 3.4 per 100 patient-years, which was plausibly lower than that of the non-dialysis population [28]. In the present study that exclusively enrolled hemodialysis patients, CVA events were also rare. In support of our finding, a recent systemic review showed that, for AF patients who were on hemodialysis, warfarin did not reduce the risk of stroke, but rather increased the risk of bleeding [29]. Indeed, the data from DOPPS also showed that in AF patients who were on hemodialysis and were > 75 years of age, the use of warfarin was associated with increased risk of stroke [28]. Altogether, these findings suggest that the use of anti-coagulation therapy should be reconsidered in ESRD patients with AF.

In our study, echocardiography measurements, including systolic function and degree of LVH were similar between AF and non-AF patients. While more AF patients were diagnosed as having HF, the EF measured by echocardiography was not significantly different from non-AF patients. One explanation may be that the AF patients experienced more diastolic heart failure with preserved EF. Nevertheless, due to the reason that atrial emptying/contraction ratio was not measurable in AF patients, it is difficult to evaluate the degree of diastolic dysfunction this group of patients.

In non-dialysis patients who experienced AMI, AF is a common complication associated with adverse outcomes [30, 31]. Also, non-dialysis patients with pre-existing AF have increased risk of AMI [32]. In the present study, significantly higher risk of CV death was noted in incident hemodialysis patients with AF, suggesting a similar risk imposed by AF in dialysis population.

AF resulted from electrophysiological abnormalities of atrial tissue, which was stimulated by several pathogenic factors such as systemic inflammation [33,34]. In accordance with this point of view, previous studies also showed that in patients with severe sepsis, new-onset or precipitated AF are frequently seen and are associated with adverse outcomes [35, 36]. On the other hand, it had also been reported that AF was associated with increased mortality in patients with pneumonia [37, 38]. The present study showed that in incident ESRD patients, AF significantly associated with sepsis-related death and that CRP was associated with risk of mortality. Both findings supported the role of systemic inflammation on the pathogenesis of AF.

The limitations of the present study included retrospective design with various uncontrolled factors, relatively small sample size, unavailable information on volume status and heart rate on the time of echocardiography. On the other hand, the strengths of our study include complete laboratory data, detailed echocardiography measurements and accurately confirmed causes of death in analysis.

In conclusion, AF is an independent predictor for mortality in incident ESRD patients irrespective of age. Cause-specific analysis showed that AF was associated with cardiovascular and sepsis-related mortality in patients on maintenance hemodialysis. While AF may deteriorate HF, systolic function and degree of LVH were not significantly different in AF and non-AF patients. Finally, the association between AF and sepsis-related mortality suggest the role of systemic inflammation on the pathogenesis of AF.

## Supporting information

S1 Dataset (XLSX)
